# Supplementary figures and images for: Understanding contributors to racial and ethnic inequities in COVID-19 incidence and mortality rates
Source: PLoS One. 2022 Jan 28;17(1):e0260262. doi: 10.1371/journal.pone.0260262 (PMC8797246; doi:10.1371/journal.pone.0260262)

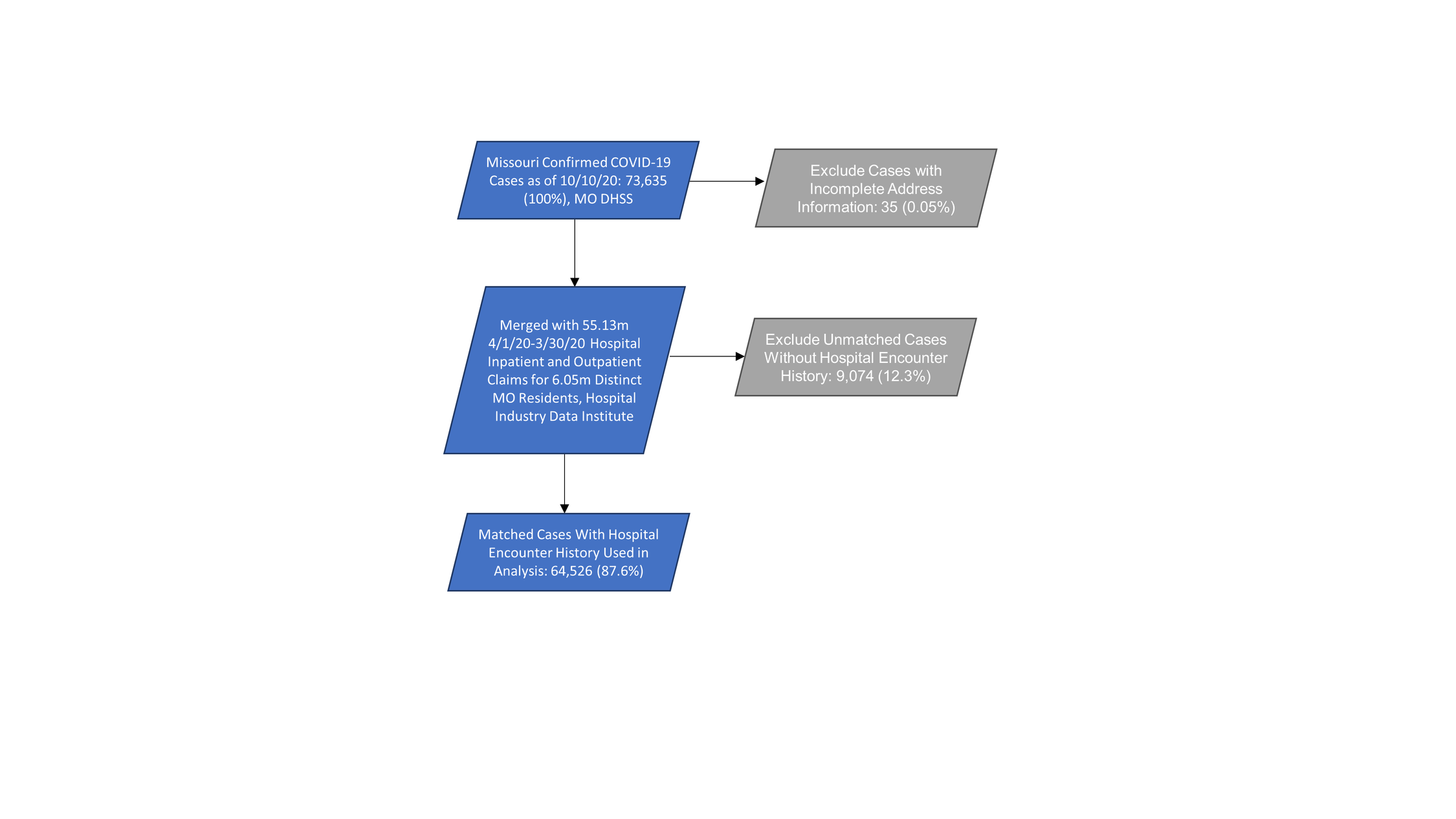

Supplement: S1 Fig — Abbreviations: MO = Missouri; MO DHSS = Missouri Department of Health and Social Services. (TIF) [file pone.0260262.s001.tif]
