# Supplementary material for: Understanding contributors to racial and ethnic inequities in COVID-19 incidence and mortality rates
Source: PLoS One. 2022 Jan 28;17(1):e0260262. doi: 10.1371/journal.pone.0260262 (PMC8797246; doi:10.1371/journal.pone.0260262)
Supplement: S1 Table — Numbers represent hospital claims rather than unique individuals, since patients could have one race reported on one claim and a different one reported on another claim. If a patient had an even number of claims with two different races (for example, two with White and two with Asian), he or she was left in the “other” category because we could not determine which was the appropriate category. (DOCX) [file pone.0260262.s002.docx]

**Supporting Information for “Understanding Contributors to Racial and Ethnic Inequities in COVID-19 Incidence and Mortality Rates”**

Karen E. Joynt Maddox, MD, MPH;^1,2^ Mat Reidhead, MA;^3^ Joshua Grotzinger, BS;^3^ Timothy McBride, PhD;^2,4^ Aaloke Mody, MD;^5^ Elna Nagasako, MD;^6^ Will Ross, MD, MPH;^7^ Joseph T. Steensma, EdD;^4^ and Abigail R. Barker, PhD^2,4^

1. Cardiovascular Division, Washington University School of Medicine, St. Louis, MO
2. Center for Health Economics and Policy, Institute for Public Health at Washington University, St. Louis, MO
3. Missouri Hospital Association, Jefferson City, MO
4. Washington University Brown School, St. Louis MO
5. Division of Infectious Diseases, Washington University School of Medicine, St. Louis, MO
6. Honolulu, HI
7. Division of Nephrology, Washington University School of Medicine, St. Louis, MO

**S1 Table: Further Breakdown of “Other” Race Category**

Distribution of Race Reported in Hospital Claims for Individuals in the Other Race Category:

|  | Count | Percent |
| --- | --- | --- |
| Total Hospital Claims | 12,032 | 100.0% |
| Patient Race Coded Other | 4,533 | 37.7% |
| Patient Race Coded Asian | 1,888 | 15.7% |
| Patient Race Coded Unknown or Refused | 1,604 | 13.3% |
| Patient Race Coded White | 1,444 | 12.0% |
| Patient Race Coded Multiple Races | 895 | 7.4% |
| Patient Race Coded Pacific Islander/Hawaiian Native | 814 | 6.8% |
| Patients with >1 Race Reported | 680 | 5.7% |
| Patient Race Coded Black/African American | 579 | 4.8% |
| Patient Race Coded American Indian/Alaskan Native | 275 | 2.3% |

Numbers represent hospital claims rather than unique individuals, since patients could have one race reported on one claim and a different one reported on another claim. If a patient had an even number of claims with two different races (for example, two with White and two with Asian), he or she was left in the “other” category because we could not determine which was the appropriate category.
