# Supplementary material for: Understanding contributors to racial and ethnic inequities in COVID-19 incidence and mortality rates
Source: PLoS One. 2022 Jan 28;17(1):e0260262. doi: 10.1371/journal.pone.0260262 (PMC8797246; doi:10.1371/journal.pone.0260262)
Supplement: S2 Table — Abbreviations: ICC, intraclass coefficient; SDOH, social determinants of health; SE, standard error. (DOCX) [file pone.0260262.s003.docx]

**Supporting Information for “Understanding Contributors to Racial and Ethnic Inequities in COVID-19 Incidence and Mortality Rates”**

Karen E. Joynt Maddox, MD, MPH;^1,2^ Mat Reidhead, MA;^3^ Joshua Grotzinger, BS;^3^ Timothy McBride, PhD;^2,4^ Aaloke Mody, MD;^5^ Elna Nagasako, MD;^6^ Will Ross, MD, MPH;^7^ Joseph T. Steensma, EdD;^4^ and Abigail R. Barker, PhD^2,4^

1. Cardiovascular Division, Washington University School of Medicine, St. Louis, MO
2. Center for Health Economics and Policy, Institute for Public Health at Washington University, St. Louis, MO
3. Missouri Hospital Association, Jefferson City, MO
4. Washington University Brown School, St. Louis MO
5. Division of Infectious Diseases, Washington University School of Medicine, St. Louis, MO
6. Honolulu, HI
7. Division of Nephrology, Washington University School of Medicine, St. Louis, MO

**S2 Table: Impact of Additional Adjustment on ICC Associated with Race Variable**

|  | Covariance Parameter Estimate | SE | Variance | ICC associated with race variable |
| --- | --- | --- | --- | --- |
| Demographics | 0.189 | 0.156 | 3.29 | 0.054 |
| Demographics+SDOH | 0.099 | 0.084 | 3.29 | 0.029 |
| Demographics+SDOH+Clinical | 0.080 | 0.071 | 3.29 | 0.024 |

Abbreviations: ICC, intraclass coefficient; SDOH, social determinants of health; SE, standard error.
