# Supplementary material for: Understanding contributors to racial and ethnic inequities in COVID-19 incidence and mortality rates
Source: PLoS One. 2022 Jan 28;17(1):e0260262. doi: 10.1371/journal.pone.0260262 (PMC8797246; doi:10.1371/journal.pone.0260262)
Supplement: Attachment — Submitted filename: Racial Inequities in COVID PLOS ONE R1 Response 8-14-21.docx [file pone.0260262.s004.docx]

Understanding Contributors to Racial and Ethnic Inequities in COVID-19 Incidence and Mortality Rates: Response Letter

**Editor**

|  | Comment | Response | Location of edit |
| --- | --- | --- | --- |
|  | There is inconsistent capitalization of “white” within text and Fig. 3 caption. | Thank you for pointing this out. We have capitalized all racial designators within the manuscript per AMA guidance. | Throughout |
|  | I suggest adding data labels to the Fig. 3 columns to be consistent with Fig. 1. | Fig 1 has been removed, but Fig 3 matches the new Fig 1 and Fig 2. Thank you for this suggestion. |  |
|  | Confirm that the PLOS ONE guideline on Acknowledgements has been followed (link below):  <https://journals.plos.org/plosone/s/submission-guidelines#loc-acknowledgments> | We have done so. |  |
|  | Please verify references follow PLOS ONE formatting requirements. For example, all journal names must be abbreviated using the NCBI database.  Please refer to the following link:  <https://journals.plos.org/plosone/s/submission-guidelines#loc-references> | Thank you for pointing this out. We have updated the reference list to reflect Vancouver formatting guidelines. | References |

**Reviewer 1**

|  | Comment | Response | Location of edit |
| --- | --- | --- | --- |
| General comments | The authors present a well written study of racial/ethnic inequities in COVID-19 incidence and mortality. The use of state-wide data is a major strength of this manuscript and the discussion of observed disparities is generally thoughtful. | We appreciate the very helpful comments provided by the reviewer! |  |
| Major comments: Analysis | The analysis of case distribution by race/ethnicity (fig 1) does not take advantage of individual-level information on race/ethnicity and instead only presents COVID-19 case rates by ZIP-code level %Black/African American. I believe you have the data necessary to examine case rates by individual-level race/ethnicity and should present this in addition to (or in lieu of) the current analysis. This would more directly answer the first part of the question set out in the Introduction: whether the case rates are different by race/ethnicity or if among those who are diagnosed with the disease, the mortality rate differs by race/ethnicity, or both. | We appreciate this comment and apologize for any confusion. We analyzed rates by ZIP among all cases which could be linked to address (99.1% of cases), and by race/ethnicity among cases which could be linked to medical records, from which we ascertained individuals’ race and ethnicity (87.7% of cases).  However, as we agree this is potentially confusing, and only focuses on one racial group, we have removed the ZIP-level analyses and now present only the linked patient-level analyses in the manuscript. | Methods section, Statistical Analysis subsection and Results |
| Major comments: Discussion | The Discussion is generally well-written and thoughtful, but primary focuses on the results for the NH Black/African American group and does not provide a discussion for the NH Other and Hispanic groups, for which elevated risks were also observed. | Thank you for raising this point. We have added text to the discussion section regarding these groups. | Discussion |
| Major comments: Discussion | The “NH Other” group had ORs higher than the NH Black group in the fully adjusted model. The authors should provide more discussion here. Is there any other information of who comprises this “Other” group (e.g., Asian, Pacific Islanders, American Indians)? What hypotheses do they have for the observed results for this group? | We agree that this is an important group and have provided additional information on their makeup in the results section and appendix. Individuals included in the Other Race category included six individual race categories (American Indian, Asian, Pacific Islander, Other, Multiple Races, and Unknown or Refused), in addition to individuals with multiple races reported in the hospital claims data where no dominant mode was detected (e.g. an individual with two claims, one coded White and one coded Asian). The largest subset of this group (about a third) was actually coded “other” while the most prevalent other race was Asian. We have also added commentary about this group to the discussion and limitations section. | Limitations, lines 564-584 |
| Major comments: Discussion | The mortality rate among Hispanic cases was elevated but was attenuated in further adjusted models. How do these results compare with the literature? Hispanics are large diverse group, with many different countries of ancestry. Are there additional data on the Hispanic individuals in these data or in Missouri in general that could inform why different results were (or were not) observed for these analysis (e.g., Hispanic origin, immigration status, etc.)? | This is an interesting point, and one on which we are glad to comment. We have added text around the Hispanic population in Missouri to the discussion section. We do not have specific information on our patients regarding their specific country of ancestry or immigration status, but agree that these factors may play a role in the results we observed. This is now discussed in the limitations as well. | Discussion |
| Other comments: Line 133 | What is meant by “race/ethnicity risk factors”? Do you mean race/ethnicity as categorical variables, or factors that vary with race/ethnicity? Please specify. | We apologize for the confusing terminology and have edited this sentence. | Introduction, line 132 |
| Other comments: Lines 155-158/Fig. 1 | Add confidence intervals for effect estimates to the figure and include in the text for effect estimates stated (i.e., 870.4 and 1,877.9). | We removed Figure 1 and its associated text per comments above. | Results |
| Other comments: Fig. 1 | Results for mortality per 100,000 population (grey bars in Fig 1) are not mentioned in the text (neither in the Results section or in the Statistical Analysis description). Include this in the text in these two sections. | Our apologies. We removed the ZIP-level analyses per the earlier suggestion. | Results |
| Other comments: Lines 233-234/Fig. 3 | If possible with this type of analysis, add confidence intervals for effect estimates to the figure and include in the text for effect estimates stated (i.e., 1.6, 2.4, etc.). | Thank you for this comment. This figure shows estimates from fixed (predicted) and random (expected) effects scaled by the observed mortality rate. The bars are P/E*obs for each group and model. Because the inputs for each group are from two separate models, there isn’t a straightforward way to get confidence estimates. Some groups, for example Yale CORE (a research group that manages a number of government contracts for quality measures) sometimes bootstraps CIs for P/E ratios in hierarchical generalized linear models, but that takes a lot of machine time (1000 iterations). More importantly, other literature suggests there is no established method for estimating CI’s for PE ratios, see page 8 in below: <https://www.sas.com/content/dam/SAS/support/en/sas-global-forum-proceedings/2020/4697-2020.pdf>  One option would be to change the figure showing a random effects model without the fixed effects (patient-level, neighborhood, etc.), which is a simpler model that does allow for regular computation of confidence intervals, but then our estimates of the contribution of the various components of risk are not feasible. For these reasons we have elected to leave the figure as-is but if the editor wishes us to change it out for the simpler random effects model we would be glad to do so. |  |
| Other comments: Lines 332-333 | The strategies listed all relate to proximal intervention strategies (i.e., closer to the individual-level—using PPE, taking sick leave, getting tested). Earlier in the Discussion you provided very clear statements about how racism and discrimination and social determinants of health may have affected COVID-19 outcomes. As such, you could include in this sentence some potential interventions targeting these more distal factors related to health. | Thank you for this suggestion. We have added interventions targeting these more distal / population-level factors and agree these are important mechanisms and interventions to consider. | Discussion, lines 592-594 |

**Reviewer 2**

|  | Comment | Response | Location of edit |
| --- | --- | --- | --- |
| Figure 1 | This is an excellent figure. However, graphically, it does not clearly show that not only are there increased case number and deaths in zip codes with higher percentage of Blacks, but also the percentage of deaths among patients diagnosed with covid increases too. | Thank you. In response to another reviewer we have removed the ZIP-level analyses. We now show case and mortality rates by race/ethnicity in a figure that parallels the mortality figure. |  |
| Figure 2 | It is interesting that there is such a discrepancy between Hispanic population and the other minorities. What do the authors think accounts for this? The data presented suggest that Hispanic and Black patients had same likelihood of being insured by Medicaid, being uninsured, and having social risk factors. Based on table 1, it would seem like the differences in outcomes was attributable to prevalence of co-morbidities? In the US, both Blacks and Hispanics have higher rates of obesity and diabetes, but in the current cohort, Hispanics appear to have lower rates of obesity and uncontrolled diabetes. Does this reflect a bias in the data capture and perhaps an issue with access to care? | This is an interesting question. In Missouri, the proportion of patients who are Hispanic is quite low, and the country of origin and immigration status differ from some other parts of the country that have larger Hispanic populations, which may explain some of the discrepancies. However, we agree that if patients lacked access to care, they might not have comorbidities coded as readily as those who had had prior interactions with the health care system, or might not be in the sample at all. This is an important limitation and we have added it to the limitations section. | Limitations, lines 564-584 |
| Lines 206-211 | White patients had lower levels of change in travel patterns – could this also suggest less cautious behavior, or reflect differences in “essential worker” status? | Thank you for bringing this up. This was confusingly reported on our end – white patients actually had higher levels of change in the direction one would think (same or greater time at home during pandemic) and we have clarified this in the table. | Table 1 |
|  | Did the authors consider the effect of rural location or distance from a major health care center? Those factors may also impact access to care. | This is a great point – certainly rural communities are at high risk of lacking access to care, both in terms of routine care and also in terms of major medical centers in the context of COVID. We have added this to the discussion. | Discussion, lines 509-518 |

**Reviewer 3**

| Line 25 | Although the authors report a 87% match rate, there were still 9000 patients who were not matched. Based on the methods, these unmatched patients did not have a hospitalization in the prior 2 years, which suggests that they were very healthy (did not require hospitalization) or did not have access to necessary medical care. | We agree that the unmatched patients likely differ from the matched ones in key ways, most importantly in their overall level of health. We have added this to the limitations section and appreciate you bringing this up. | Introduction, line 125 |
| --- | --- | --- | --- |
| Line 32 | Unclear what 99.1% represents - % of total COVID cases +/- address info? | Apologies; we have rewritten this section to be clearer. | Abstract, line 32 |
| Line 37 | COVID-19 | We have updated this. | Abstract section, page 2, line 35 |
| Line 40 | Non-Hispanic other race | We have updated this. |  |
| Line 40 | Throughout Manuscript Use COVID-19, not just COVID | Thank you for pointing this out. We have edited the manuscript to reflect consistent language. | Throughout |
| Line 83 | Do you ever use MPID again? If not, do not need to define abbreviation | Thank you for pointing this out; we have removed the abbreviation. | Methods section, Data subsection, line 84 |
| Lines 98-102 | References for sources | We have added references. | Methods section, Data subsection, lines 168-171 |
| Lines 105 | (March-June, 2020) | We have updated this. | Methods section, Data subsection, line 174 |
| Line 110 | Place Non-Hispanic Other at the end of the list, since it is a catch all category | We have updated this. | Methods section, Predictor and Outcomes subsection, line 180 |
| Line 129 | Inconsistent race terminology throughout paper | Thank you for pointing this out. We have edited the manuscript to reflect consistent language. | Throughout |
| Line 154 | Line glosses over important details – how many total cases, how many with complete address information? Methods cites 73,600 cases. Need a flow diagram, and more transparent text here and in methods to understand analytic denominator of study. | We have clarified the analytic sample, and now show the total number of cases as well as those which matched to prior hospital utilization data. We have added a flow diagram to the supplement. | Results section, Case characteristics subsection, lines 239-253 |
| Line 155 | Provide total number of deaths, and overall case fatality rate. | We have added this information. | Results section, Case characteristics subsection, lines 239-253 |
| Fig. 1 | Unclear what n=xx represents. Clarify legend. | We have removed this figure. |  |
| Fig. 2 | X axis labels crowded and difficult to read. | We have spread out the axis labels. |  |
| Table 1 | Age needs units, e.g., year, which implies continuity. | We have updated this. | Table 1 |
| Table 1 | Add number of deaths row | We have added this. | Table 1 |
| Table 1 | Race terminology does not match rest of the paper, e.g., Other/Unknown – harmonize. | Thank you for pointing this out. We have edited the manuscript to reflect consistent language. | Throughout |
| Table 1 | Why were quintiles used in table/model? No mention in Methods why/how you collapsed categories. | We have added this to the methods section and apologize for the lack of clarity. | Methods |
| Table 1 | Do you mean to say 32.6% of people spent less time at home in 2020 vs 2019, or that 67.4% spent more time at home during COVID-19. | The reviewer is correct, but we agree that’s a confusing way to say it and have inverted these numbers to be clearer. We have also changed the text, where we got this backwards, and appreciate the reviewer catching our error. | Table 1 |
| Line 179 | “Matched Patients” is a confusing term in the title. Remove term, flow chart would clarify your analytic sample. | We apologize for the confusion and agree that matched patients is a confusing term. We have changed the terminology to “merged” patients to be clear that we are not matching from a separate sample. | Results section, Case Characteristics subsection, line 240 |
| Line 199 | Limit P value to < 0.001 | We have made this change. |  |
| Lines 201, 206 | Starting consecutive paragraphs with “In terms of…” is awkward – change both. | Thank you for your suggestion. We have updated the diction to read “Regarding” and “For,” respectively. | Results section, Clinical Outcomes subsection, lines 344, 349 |
| Figure 3 | Narrow brackets showing bars that you are comparing – appears that you are comparing blue and orange bars, but does not line up exactly | We have updated the figure brackets for clarity. | Figure 3 |
| Table 2 | Age needs units label, e.g., years. Why was age modeled as a continuous linear variable? Many other models find it is not linear, and use categories to define age risk, e.g., King PLOS One 2020, or splines, e.g., Mathur Lancet 2021. | We agree there are many ways to model age; in this case because we were relying on a standard risk adjustment model that uses age as a continuous variable we followed that approach for fidelity to the model. We have added the label and appreciate the suggestion to do so. | Table 2 |
| Line 251 | “… white or Hispanic…”? | Thank you – clarified. | Discussion, line 435 |
| Line 252 | You are now being vague about a precise terminology for race category used throughout your paper. | Thank you – clarified. | Discussion, lines 434-437 |
| Lines 276-291 | After stating that the reasons for this are unclear, the authors charge ahead into a discussion about racism as the etiology. They never mention the term disparities a description, instead jumping to racism as a causal explanation. The underlying theme seems to be worse outcomes for Blacks = racism. | We agree that what we have provided is descriptive information, and that racism is only one potential underlying mechanism. We have edited the discussion to include a broader range of etiologies, though we have retained racism as a key point since we believe this to be a foundational issue underlying inequities in outcomes between Black, Other race, and white patients. | Discussion, lines 464-466 |
| Line 313 | Add Rentsch 2021 PLOS Medicine paper for another take on VA race issue. | Thank you; we have done so. | Discussion, line 535 |
| Line 315 | Do you mean “laboratory data” when you state “clinical data”? Rentsch paper did not use laboratory data, but rather pre-existing diagnoses, the same as your analysis. | Thank you; it was the Ioannu study we referenced, but we have removed the reference to laboratory data since we now cite multiple VA papers. | Discussion, line 551 |
| Line 327 | Line implies that structural racism is casual in this paper, which the authors do not prove, and admit there are other explanations. Suggest disparities – a descriptor, rather than racism, a hypothesized cause. | Thank you; we have edited the line in question. | Discussion, limitations subsection, line 564 |
| Line 327 | What about patients dropped who could not be matched? 2018 census data, with population shifts in the interim? 2015 Area Depravation Index data, with changes over the last 5 years? Those without cell phones? Lack of outpatient diagnoses to determine comorbidities. How reliable is your death data? | We now include mention of the dropped patients in the limitations section and appreciate the suggestion to do so. We have included these other factors as limitations as well. We do have outpatient diagnoses, but only those that occur at hospital outpatient settings (ie specialty clinic located at a hospital) and not those at non-hospital clinic settings (ie independent primary care practice). | Discussion, limitations subsection |
|  | Throughout the document, authors are inconsistent about the use COVID vs COVID-19. Change all COVID to COVID-19. | Thank you for pointing this out. We have edited the manuscript to reflect consistent language. | Throughout |
|  | Reference 22 has odd format, prune. | Thank you for pointing this out. We have edited the reference to the formatting requirements. | References, Lines 416-420 |
